# A Comparison of hs-CRP Levels in New Diabetes Groups Diagnosed Based on FPG, 2-hPG, or HbA1c Criteria

**DOI:** 10.1155/2016/5827041

**Published:** 2015-12-28

**Authors:** Yildiz Tutuncu, Ilhan Satman, Selda Celik, Nevin Dinccag, Kubilay Karsidag, Aysegul Telci, Sema Genc, Halim Issever, Jaakko Tuomilehto, Beyhan Omer

**Affiliations:** ^1^Division of Endocrinology & Metabolism, Department of Internal Medicine, Istanbul Medical Faculty, Istanbul University, 34093 Istanbul, Turkey; ^2^Department of Clinical Biochemistry, Istanbul Medical Faculty, Istanbul University, 34093 Istanbul, Turkey; ^3^Department of Public Health, Istanbul Medical Faculty, Istanbul University, 34093 Istanbul, Turkey; ^4^Centre for Vascular Prevention, Danube-University Krems, 3500 Krems, Austria; ^5^Department of Chronic Disease Prevention, National Institute for Health and Welfare, 00271 Helsinki, Finland; ^6^Diabetes Research Group, King Abdulaziz University, Jeddah 21589, Saudi Arabia

## Abstract

Fasting plasma glucose (FPG) and hemoglobin A1c (HbA1c) have been used to diagnose new-onset diabetes mellitus (DM) in order to simplify the diagnostic tests compared with the 2-hour oral glucose tolerance test (OGTT; 2-hPG). We aimed to identify optimal cut-off points of high sensitive C-reactive protein (hs-CRP) in new-onset DM people based on FPG, 2-hPG, or HbA1c methods. Data derived from recent population-based survey in Turkey (TURDEP-II). The study included 26,499 adult people (63% women, response rate 85%). The mean serum concentration of hs-CRP in women was higher than in men (*p* < 0.001). The people with new-onset DM based on HbA1c had higher mean hs-CRP level than FPG based and 2-hPG based DM cases. In HbA1c, 2-hPG, and FPG based new-onset DM people, cut-off levels of hs-CRP in women were 2.9, 2.1, and 2.5 mg/L [27.5, 19.7, and 23.5 nmol/L] and corresponding values in men were 2.0, 1.8, and 1.8 mg/L (19.0, 16.9, and 16.9 nmol/L), respectively (sensitivity 60–65% and specificity 54–64%). Our results revealed that hs-CRP may not further strengthen the diagnosis of new-onset DM. Nevertheless, the highest hs-CRP level observed in new-onset DM people diagnosed with HbA1c criterion supports the general assumption that this method might recognize people in more advanced diabetic stage compared with other diagnostic methods.

## 1. Introduction

The relation between chronic subclinical low-grade inflammation and insulin resistance (IR) has long been known [1, 2]. IR is the major contributor and mediating factor in the development of type 2 DM (T2DM) along with concomitant hypertension (HT) and cardiovascular disease (CVD) [3, 4]. The relationship between the development of DM and some markers of inflammation such as C-reactive protein (CRP), IL-6, fibrinogen, and PAI-1 has been described previously. Serum concentration of CRP increases in both impaired glucose tolerance (IGT) and overt T2DM [3, 5–10]. On the other hand, some studies reported that elevation of CRP is an indicator of development of T2DM [11].

Compared with the conventional OGTT (2-hPG) as recommended by WHO as gold standard, fasting plasma glucose (FPG) and HbA1c are more convenient, simpler, and cost-effective diagnostic methods that are currently in use for the diagnosis of T2DM [5, 12–17]. However, each test recognizes people with different metabolic features and groups who may be diagnosed by different tests but do not overlap substantially. While high postchallenge plasma glucose is a strong predictor of CVD, fasting glucose is not an independent predictor of CVD [18]. Consequently, further tests that will strengthen the diagnosis of DM are needed.

To the best of our knowledge, there is no previous report specifically comparing the role of hs-CRP in people with newly diagnosed DM with the criteria based on the 2-hPG, FPG, and HbA1c. Therefore, the aim of this study was to identify the optimal cut-off points of hs-CRP in new-onset (previously undiagnosed) people with DM diagnosed based on the current 2-hPG, FPG, and HbA1c diagnostic criteria. In this study, hs-CRP results obtained from a nationally representative population-based survey are being reported.

## 2. Material and Methods

Data derived from “The Turkish Epidemiology Survey of Diabetes, Hypertension, Obesity and Endocrine Diseases (TURDEP-II),” a population-based study, which was included randomly assigned 26,499 adult people from 270 urban and 270 rural centers. The field survey was performed between January and June 2010, with a participation rate of 85%. The study protocol was described elsewhere [19]. A written informed consent was obtained from each participant. The study was approved by the local ethical board (Istanbul Medical Faculty Ethical Committee, 16.4.2008/699).

People with known DM or other systemic diseases who had hs-CRP levels of 10 mg/L (95.2 nmol/L) or above were excluded from this study due to a possible infection. Final assessments included 21,485 (63.6% women) participants. All biochemical tests including glucose, insulin, and lipid profile were measured in fasting blood samples using Roche Diagnostics Modular Autoanalyzer System (Roche Diagnostics, Germany) in the Central Biochemistry Laboratory of Istanbul Medical Faculty. Concentration of hs-CRP was analyzed by immunoturbidimetric assay (Roche/Hitachi 912, Modular P analyzers: ACN 210; CRPL3 Tina-quant C-reactive protein Gen. 3) and HbA1c by turbidimetric inhibition immunoassay; both the system and the laboratory have been regularly certified (Roche Diagnostics TQ HbA1c Gen. 3; NGSP Certificate of Traceability; September 2010-2011).

A detailed medical history of each participant was obtained, and measurements of anthropometry (height, weight, waist, and hip circumference) and systolic and diastolic blood pressure (SBP, DBP) were done. Body mass index (BMI), HOMA-IR (= fasting glucose × fasting insulin/405), and non-HDL-cholesterol (= total cholesterol − HDL-cholesterol) were calculated accordingly. Glomerular filtration rate (eGFR) was estimated using “Chronic Kidney Disease Epidemiology Collaboration” CKD-EPI equation [20].

### 2.1. Statistical Analysis

The mean values of continuous variables were compared using *t*-test. Risk factors for DM were evaluated using chi-square test; mean values by sex were compared using nonparametric Mann-Whitney *U* test. Homogeneity of variance and normal distribution of variables were tested by Kolmogorov-Smirnov test. Pearson correlation coefficients (*r* values) were calculated to assess the association between hs-CRP and other laboratory parameters. Mean levels of hs-CRP in each of the new DM groups were further compared using univariate analysis after being adjusted for age, BMI, smoking and alcohol drinking, and SBP and DBP. An optimum cut-off point of hs-CRP was estimated using the receiver operating characteristic (ROC) curve and area under the curve (AUC) with 95% CIs was calculated for each of the new DM groups that were diagnosed with FPG, ≥126 mg/dL (7.0 mmol/L), 2-hPG: ≥200 mg/dL (11.1 mmol/L), or HbA1c: ≥6.5% (48 mmol/mol) [13, 17]. Optimum cut-off points of hs-CRP were defined for men and women, separately. As the raw data not normally distributed when log transformed, we obtained a square root transformation (sqrt) of the hs-CRP levels, and after defining the cut-off points we recalculated the squares.

Data were analyzed using SPSS for Windows (version 21.0; SPSS/IBM, Chicago, IL). A *p* value less than 0.05 was considered statistically significant.

## 3. Results

### 3.1. Main Characteristics of the Study Population

Demographic characteristics and laboratory findings of women and men in TURDEP-II study are presented in Table 1. In brief, men were significantly older and had higher mean weight, waist circumference, SBP, DBP, triglycerides (TG), HDL-cholesterol, and non-HDL-cholesterol values than women. Women had significantly higher BMI, hip circumference, heart rate, FPG, HbA1c, 1-hPG, 2-hPG, HDL-cholesterol, and eGFR values than men. Median (interquartile range, IQR) concentration of hs-CRP in women was significantly higher than in men (women, 1.85 [3.09] mg/L [17.6 [29.4] nmol/L], versus men, 1.47 [2.33] mg/L [14.00 [22.2] nmol/L], *p* < 0.001). Total serum cholesterol, fasting serum insulin, and HOMA-IR values did not differ between men and women (Table 1).

### 3.2. Correlation Analysis of hs-CRP

There was a positive correlation between hs-CRP levels and age, BMI, waist, hip, SBP, DBP, pulse, FPG, HbA1c, 1-hPG, 2-hPG, TG, non-HDL-cholesterol, and HOMA-IR; and there was a negative correlation with HDL-cholesterol and eGFR. When we repeated the analysis after controlling for HT, age, sex, smoking and alcohol use, BMI, and waist circumference, the positive correlations between hs-CRP levels and HbA1c, 1-hPG, 2-hPG, TG, non-HDL-cholesterol, eGFR, and HOMA-IR and the negative correlation with HDL-cholesterol and creatinine remained significant (Table 2).

### 3.3. Mean hs-CRP in Newly Diagnosed DM Groups and Sex Difference

Among people with new DM, the highest hs-CRP level was in the group detected by HbA1c criterion (hs-CRP median [IQR]; HbA1c: 3.45 [3.82] mg/dL, 32.9 [36.4] nmol/L; 2-hPG: 2.7 [3.14] mg/dL, 25.4 [29.9] nmol/L; and FPG: 2.4 [3.0] mg/dL, 22.4 [28.5] nmol/L, data not shown).

High sensitive CRP level was significantly higher in women than men with newly diagnosed DM groups based on 2-hPG, FPG, and HbA1c criteria. Among the newly diagnosed DM groups, the median [IQR] level of hs-CRP was highest in those detected with HbA1c in both genders (HbA1c-group: women: 4.0 [4.1] mg/dL, 38.4 [39.1] nmol/L; men: 2.7 [3.1] mg/dL, 25.9 [29.1] nmol/L; FPG group: women: 3.3 [4.1] mg/dL, 31.1 [39.3] nmol/L; men: 2.4 [3.0] mg/dL, 22.4 [28.5] nmol/L; and 2-hPG group: women: 2.8 [3.3] mg/dL, 26.5 [31.3] nmol/L; men: 2.4 [2.8] mg/dL, 23.3 [26.9] nmol/L).

Sex differences in hs-CRP did not change after the data adjusted with respect to age, BMI, waist circumference, and HT; the mean hs-CRP was highest in newly diagnosed patients based on HbA1c criterion in both sexes. Again, the average hs-CRP levels of women were higher than men (Table 3). In women with newly diagnosed DM based on FPG, mean hs-CRP level was comparable to those diagnosed with 2-hPG but lower than the group diagnosed with HbA1c (*p* = 0.000032). Women with newly detected DM based on 2-hPG had also lower mean hs-CRP than those detected with HbA1c (*p* < 0.000001). In contrast, men with newly detected DM based on FPG criterion had lower mean hs-CRP than those diagnosed with 2-hPG (*p* = 0.017) and HbA1c (*p* = 0.003) but mean hs-CRP levels were comparable in 2-hPG and HbA1c based new DM groups (Table 3).

### 3.4. Optimal Cut-Off Points and AUCs of hs-CRP for Newly Diagnosed DM Groups

**Figure 1:**
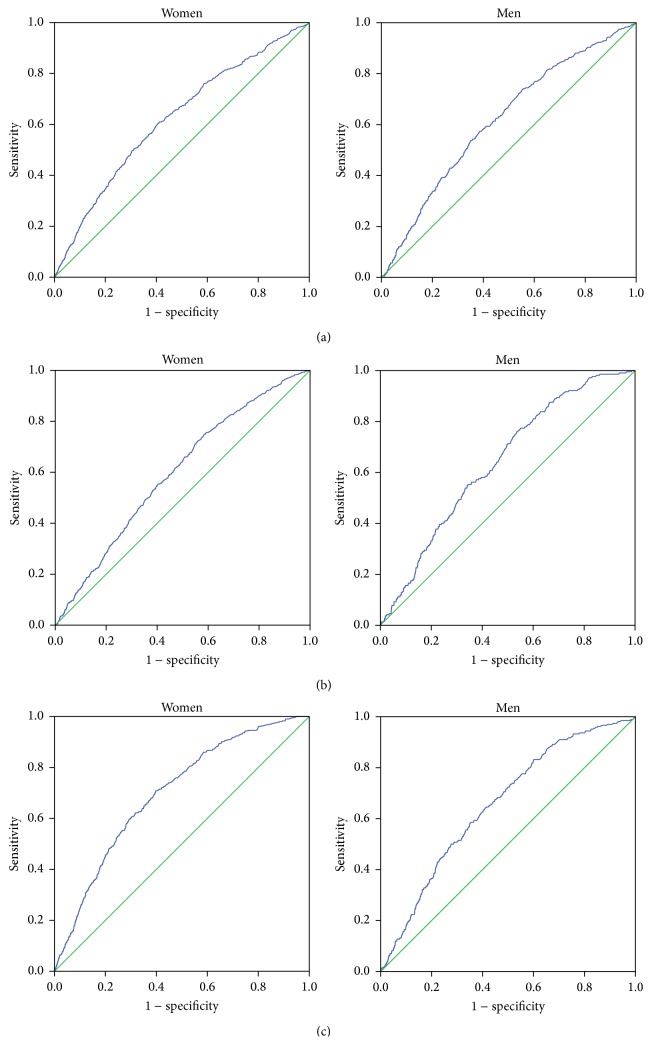
ROC curves in women and men new-onset DM groups diagnosed with (a) FPG, (b) 2-hPG, and (c) HbA1c criteria (ROC: receiver operating characteristic curve; FPG: fasting plasma glucose; 2-hPG: 2-hour plasma glucose during OGTT; HbA1c: hemoglobin A1c).

The specificity and sensitivity of the optimal cut-off points for hs-CRP to detect DM in women were for the FPG group, 60% and 57% for 2.5 mg/L (23.6 nmol/L), 2-hPG group, 60% and 54% for 2.1 mg/L (19.7 nmol/L), and 65% and 64% for HbA1c group, 2.9 mg/L (27.5 nmol/L). In men the corresponding specificity and sensitivity values were as follows: FPG group: 60% and 57% for 1.8 mg/L (16.9 nmol/L); 2-hPG group: 60% and 57% for 1.8 mg/L (16.9 nmol/L); and HbA1c group: 65% and 60% for 2.0 mg/L (19.0 nmol/L) (ROC curves Figure 1 and Table 4). The largest AUC value for hs-CRP to detect DM was found in women and men when using HbA1c (women: 0.700; men: 0.656).

The positive and negative predictive values (PPV and NPV) corresponding to the above-mentioned cut-points of hs-CRP in women were calculated as 58% and 59% for FPG, 57% and 58% for 2-hPG, and 64% and 65% for HbA1c and in men were 58% and 59% for FPG, 58% and 59% for 2-hPG, and 61% and 61% for HbA1c. The best results for PPV and NPV were obtained by the HbA1c method.

## 4. Discussion

Our current population-based study identified 1,727 people with newly diagnosed DM based on at least one of the three methods. However, the people identified to have DM were substantially different for each of the three methods. In other words, the concordance rate for DM among the different methods of glycaemia testing was low. There are probably several reasons for this discrepancy. Characteristics of the people with DM and their risk factors may vary by the method used for the detection of DM.

Several studies have suggested that inflammation is associated with IR that takes part in the pathogenesis of T2DM and atherosclerotic disease [1–4, 6]. Environmental factors such as infections, overnutrition, and lack of physical activity are believed to contribute serum CRP levels although the mechanism is not properly understood. On the other hand, hyperglycaemia per se may induce inflammation and this may enhance the development of DM [21, 22].

Festa et al. demonstrated that people who developed DM (detected by an OGTT) had higher baseline serum CRP levels than those who did not develop DM [23]. There was a linear increasing trend in the incidence of DM as the baseline CRP quartile increased [23]. In Pizarra prospective study, people with baseline hs-CRP ≥3 mg/L (≥28.6 nmol/L) developed DM [24]. In our previous report, we demonstrated a linear increasing trend for hs-CRP levels from normal glucose tolerance through impaired fasting glucose (IFG), IGT, and new DM [19].

A recent meta-analysis including 18 prospective studies demonstrated that high baseline CRP levels associated with future T2DM diagnosed based on FPG and/or 2-hPG criteria [25]. All these findings support the chronic low-grade inflammation hypothesis in the development of DM.

In our study, we found a positive correlation between hs-CRP levels and all glycaemia and IR parameters. However, after adjustment for age, sex, smoking, BMI, waist, and HT, positive correlations were maintained with HbA1c, 1-hPG and 2-hPG, fasting insulin, and HOMA-IR but not with FPG. In their later report Festa et al. stated that postchallenge glucose but not FPG was strongly correlated with baseline CRP [26]. Other studies have also shown an association between CRP and DM, which remained significant after adjusting for BMI or other covariates [25]. Our findings and others suggested that adiposity is not sufficient to explain the relationship between high levels of inflammatory markers and increased DM risk. The predictive value of hs-CRP did not seem to be fully independent of obesity in several [3, 23, 24, 27, 28] but not all studies [7, 9, 10, 22].

Our results showed that among people with new DM the highest hs-CRP levels were obtained in those identified with the HbA1c criterion. HbA1c elevation at diagnosis is an indication of overt DM, but in a more advanced stage compared with new-onset DM detected with the FPG or 2-hPG criteria. As we did show a positive correlation between hs-CRP and HbA1c, it has been reported that people with DM with poorer glycaemic control had higher CRP levels [8]. Some commonly used medications like aspirin and statin may synergistically reduce serum CRP concentrations [29, 30]. In the present study, we excluded people with known systemic disease and those who self-reported regularly using such medications; however, we may not ascertain all people using aspirin and/or statin.

Aronson et al. reported that CRP levels among middle-aged people were higher in those with DM and IFG when compared with the healthy subjects [21]. Similar to our findings showing women had higher hs-CRP levels than men regardless of having DM or not, the ADOPT investigators have reported that hs-CRP levels in women were higher than in men in both with and without the metabolic syndrome (MS) [31]. They reported a positive correlation between hs-CRP and HbA1c, BMI and HOMA-IR and the number of MS components in people with new DM [31]. In Women's Health Study, the incidence of DM was four times higher in women with hs-CRP levels in the upper quartile in comparison with those with hs-CRP levels in the lowest quartile [32].

Wu and coworkers reported that high levels of hs-CRP were correlated with high levels of HbA1c and FPG in men and with only FPG in women [33]. A similar finding was also reported by Festa et al. [3]. Other studies reported strong correlations between hs-CRP and fasting insulin [7], HOMA-IR [28], and FPG [21, 34] and an inverse correlation with CRP and HDL-cholesterol [3, 21]; all were confirmed in our study.

We estimated the optimal cut-off hs-CRP and AUC-CRP with 95% CIs for DM for each of the three diagnostic methods separately. To the best of our knowledge, this is the first study aiming at determining hs-CRP cut-off points indicating new DM compared with each of the three methods assessing hyperglycaemia. The highest cut-off point for hs-CRP was obtained with HbA1c based new DM detection compared to FPG and 2-hPG methods. In fact we previously showed that new DM group detected with HbA1c has a more advanced metabolic disorder (higher BMI, waist, blood pressure, non-HDL-cholesterol, triglycerides, and insulin but lower HDL-cholesterol) than other new DM groups detected with FPG or 2-hPG [35].

den Engelsen et al. attempted to find a cut-off point for hs-CRP that would indicate the presence of the MS [36]. If hs-CRP cut-off point was set at 3 mg/L (28.6 nmol/L), the sensitivity and specificity were 72% and 37%; at this point PPV and NPV were 42% and 67% [36]. In another study performed in a Japanese population, a cut-off point of hs-CRP of 0.65 mg/L (6.2 nmol/L) for FPG 100 mg/dL (5.6 mmol/L) or higher was capable of defining the MS with a 100% sensitivity and 77% specificity in women and with a 65% sensitivity and 63% specificity in men [37]. In our study the optimum cut-point of hs-CRP of 2.5 mg/L (23.6 nmol/L) in women and 1.8 mg/L (16.9 nmol/L) in men for FPG ≥126 mg/dL (7.0 mmol/L) or higher was capable of defining the new DM with a 60% sensitivity and 57% specificity in both genders. The optimum cut-point for HbA1c 6.5% and over was 2.9 mg/L (27.5 nmol/L) in women with a 65% sensitivity and 64% specificity and 2.0 mg/L (19.0 nmol/L) in men with a 60% sensitivity and 62% specificity, and the largest AUC value for hs-CRP to detect new DM was found in women and men when using HbA1c (women: 0.700; men: 0.656). These high cut-off points of CRP may be related with the more advanced diabetic state as compared to FPG or 2-hPG based detected cases.

One of the greatest strengths of the present study is its national representative sampling with a large sample size and wide age range. In addition, this is the first study where all three currently proposed methods (2-hPG, FPG, and HbA1c) were used to define DM and each of them was compared with the inflammation marker, hs-CRP. The major limitations are the cross-sectional design and somewhat higher participation rate in women, controlled in the data analyses.

In brief, an hs-CRP level ≥1.8 mg/L (16.9 nmol/L) generally detects more than half of the people with new DM. External validation of our findings needs to be carried out in additional studies in other populations with reasonably large sample size before these findings can be generalized.

## 5. Conclusions

Our results revealed that hs-CRP may not further strengthen the diagnosis of new-onset DM. However, the highest hs-CRP among people with new DM was found in those identified with the HbA1c criterion. This suggests that high HbA1c may recognize new DM cases at a more advanced stage than FPG or 2-hPG in an OGTT. Clinical implications of this finding deserve further evaluation. It would be important to find out if people with newly detected DM with high hs-CRP require a more intensive therapy than those with low hs-CRP.

## Figures and Tables

**Table 1:** Clinical characteristics and laboratory findings of TURDEP-II study population^*∗*^.

Parameter	Women (*n* = 13,676)	Men (*n* = 7,809)	*p* value
Age (year)	43 (15)	44.7 (15.6)	<0.000001
BMI (kg/m^2^)	28.6 (5.7)	27.2 (4.3)	<0.000001
Waist (cm)	91.3 (14.5)	96.4 (12.8)	<0.000001
Hip (cm)	108.6 (13.2)	105.1 (10.3)	<0.000001
SBP (mmHg)	118 (27)	120 (22)	0.000008
DBP (mmHg)	74 (13)	75 (12)	<0.000001
HR (beat/min)	79.5 (8.7)	78.3 (9.2)	<0.000001
hs-CRP (mg/L)^*∗∗*^ [nmol/L]	1.85 (3.09) 17.6 (29.4)	1.47 (2.33) 14.0 (22.2)	<0.000001
FPG (mmol/L)	5.5 (1.03)	5.48 (1.18)	0.000449
HbA1c (mmol/mol) [%]	38 (7) [5.6 (0.6)]	37 (8) [5.5 (0.7)]	0.003493
1-hPG (mmol/L)	8.9 (2.5)	8.76 (2.52)	0.000013
2-hPG (mmol/L)	7.2 (2.1)	6.34 (2.04)	<0.000001
Creatinine (*μ*mol/L)	64.0 (11.0)	82.0 (14.4)	<0.000001
Triglycerides (mmol/L)	1.4 (0.8)	1.67 (1.15)	<0.000001
HDL-cholesterol (mmol/L)	1.3 (0.3)	1.09 (0.27)	<0.000001
Non-HDL-cholesterol (mmol/L)	3.5 (1.0)	3.69 (1.01)	<0.000001
Fasting insulin (pmol/L)	56.4 (49.6)	56.9 (60.5)	0.570725
HOMA-IR	1.9 (2.9)	2.0 (3.1)	0.258439
eGFR^*∗∗∗*^ (mL/min per 1.73 m^2^) [mL/s per 1.73 m^2^]	101.0 (17.4) [0.14 (0.04)]	98.8 (16.9) [0.13 (0.04)]	<0.000001

^*∗*^mean (SD); ^*∗∗*^median (IQR); ^*∗∗∗*^CKD-EPI equation.

1-hPG, oral glucose tolerance test 1st hour plasma glucose; 2-hPG, oral glucose tolerance test 2nd hour plasma glucose; BMI, body mass index; CKD-EPI, Chronic Kidney Disease Epidemiology Collaboration; DBP, diastolic blood pressure; eGFR, estimated glomerular filtration rate; FPG, fasting plasma glucose; HbA1c, hemoglobin A1c; HDL-cholesterol, high density lipoprotein cholesterol; HOMA-IR, homeostasis model of assessment; HR, heart rate; hs-CRP, high sensitive C-reactive protein; IQR, interquartile range; SBP systolic blood pressure.

**Table 2:** Pearson correlation analysis (unadjusted and adjusted) of hs-CRP and other parameters.

Unadjusted Pearson correlation: hs-CRP versus	*r*	*p*	Controlled for age, sex, smoking, alcohol, BMI, waist, HT, and medications: hs-CRP versus	*r*	*p*
Age	0.18	<0.001	HbA1c	0.08	<0.001
BMI	0.37	<0.001	1-hPG	0.07	0.001
Waist	0.30	<0.001	2-hPG	0.07	<0.011
Hip	0.29	<0.001	Creatinine	−0.05	0.001
SBP	0.12	<0.001	Triglycerides	0.09	<0.001
DBP	0.13	<0.001	Total cholesterol	0.05	0.018
HR	0.07	<0.001	HDL-cholesterol	−0.06	0.002
FPG	0.12	<0.001	Non-HDL-cholesterol	0.07	0.001
HbA1c	0.19	<0.001	Fasting insulin	0.07	<0.001
1-hPG	0.19	<0.001	HOMA-IR	0.06	0.002
2-hPG	0.15	<0.001	eGFR	0.05	0.014
Creatinine	−0.12	0.001			
HDL-cholesterol	−0.10	<0.001			
Non-HDL-cholesterol	0.20	<0.001			
Fasting insulin	0.13	<0.001			
HOMA-IR	0.11	<0.001			
eGFR	−0.13	<0.001			

1-hPG, oral glucose tolerance test 1st hour plasma glucose; 2-hPG, oral glucose tolerance test 2nd hour plasma glucose; BMI, body mass index; DBP, diastolic blood pressure; eGFR, estimated glomerular filtration rate; FPG, fasting plasma glucose; HbA1c, hemoglobin A1c; HDL-cholesterol, high density lipoprotein cholesterol; HOMA-IR, homeostasis model of assessment; HR, heart rate; hs-CRP, high sensitive C-reactive protein; HT, hypertension; SBP, blood pressure.

**Table 3:** hs-CRP levels in women and men with newly detected DM using the FPG, 2-hPG, or HbA1c criteria^*∗*^.

Diagnostic methods	hs-CRP; mg/L [nmol/L]			
	Women		Men	
	Mean (SEM), (*n*)	95% CI	Mean (SEM), (*n*)	95% CI
FPG-DM (*n* = 477)	3.3 (0.1), (*n* = 309) [31.5 (1.3)]	3.0–3.5 [28.6–33.6]	2.5 (0.2) (*n* = 168) [23.4 (1.6)]	2.13–2.80 [20.3–26.6]

2-hPG-DM (*n* = 653)	3.2 (0.1) (*n* = 483) [30.5 (1.0)]	3.0–3.40 [28.5–32.4]	3.0 (0.2) (*n* = 170) [29.0 (1.6)]	2.7–3.4 [25.9–32.1]

HbA1c-DM (*n* = 597)	4.0 (0.1) (*n* = 356) [38.5 (1.2)]	3.8–4.3 [36.2–40.8]	3.1 (0.1) (*n* = 241) [29.8 (1.3)]	2.9–3.40 [27.2–32.4]

Post hoc comparisons	FPG-DM versus 2-hPG-DM, *p* = 0.698		FPG-DM versus 2-hPG-DM, *p* = 0.017	
	FPG-DM versus HbA1c-DM, *p* = 0.000032		FPG-DM versus HbA1c-DM, *p* = 0.003	
	2-hPG-DM versus HbA1c-DM, *p* < 0.000001		2-hPG-DM versus HbA1c-DM, *p* = 0.0695	

^*∗*^Adjusted for age, BMI, waist, and hypertension (HT). Women: *p* < 0.000001; men: *p* = 0.008.

**(a):** 

Method	Gender	AUC	SEM	*p*	95% CI
FPG	Women	0.622	0.012	<0.001	0.598–0.646
	Men	0.617	0.015	<0.001	0.588–0.646

2-hPG	Women	0.599	0.011	<0.001	0.577–0.621
	Men	0.640	0.017	<0.001	0.606–0.673

HbA1c	Women	0.700	0.012	<0.001	0.676–0.723
	Men	0.656	0.016	<0.001	0.625–0.686

**(b):** 

Method	Best cut-off points					
	Women			Men		
	hs-CRP mg/L [nmol/L]	Sensitivity	Specificity	hs-CRP mg/L [nmol/L]	Sensitivity	Specificity
FPG	2.5 [23.6]	0.60	0.57	1.8 [16.9]	0.60	0.57
2-hPG	2.1 [19.7]	0.60	0.54	1.8 [16.9]	0.60	0.57
HbA1c	2.9 [27.5]	0.65	0.64	2.0 [19.0]	0.60	0.62
